# Machine learning-based analysis of cancer cell-derived vesicular proteins revealed significant tumor-specificity and predictive potential of extracellular vesicles for cell invasion and proliferation – A meta-analysis

**DOI:** 10.1186/s12964-023-01344-5

**Published:** 2023-11-20

**Authors:** Matyas Bukva, Gabriella Dobra, Edina Gyukity-Sebestyen, Timea Boroczky, Marietta Margareta Korsos, David G. Meckes, Peter Horvath, Krisztina Buzas, Maria Harmati

**Affiliations:** 1 Department of Immunology, Albert Szent-Györgyi Medical School, Faculty of Science and Informatics, University of Szeged, 6726 Szeged, Hungary; 2 Doctoral School of Interdisciplinary Medicine, Albert Szent-Györgyi Medical School, University of Szeged, 6720 Szeged, Hungary; 3 Laboratory of Microscopic Image Analysis and Machine Learning, Institute of Biochemistry, Biological Research Centre, Hungarian Research Network (HUN-REN), Szeged, 6726 Hungary; 4 Department of Biomedical Sciences, Florida State University College of Medicine, Tallahassee, FL 32306 USA

**Keywords:** Extracellular vesicles, NCI-60, Invasion, Proliferation, Classification, Prediction, Machine learning

## Abstract

**Background:**

Although interest in the role of extracellular vesicles (EV) in oncology is growing, not all potential aspects have been investigated. In this meta-analysis, data regarding (i) the EV proteome and (ii) the invasion and proliferation capacity of the NCI-60 tumor cell lines (60 cell lines from nine different tumor types) were analyzed using machine learning methods.

**Methods:**

On the basis of the entire proteome or the proteins shared by all EV samples, 60 cell lines were classified into the nine tumor types using multiple logistic regression. Then, utilizing the Least Absolute Shrinkage and Selection Operator, we constructed a discriminative protein panel, upon which the samples were reclassified and pathway analyses were performed. These panels were validated using clinical data (*n* = 4,665) from Human Protein Atlas.

**Results:**

Classification models based on the entire proteome, shared proteins, and discriminative protein panel were able to distinguish the nine tumor types with 49.15%, 69.10%, and 91.68% accuracy, respectively. Invasion and proliferation capacity of the 60 cell lines were predicted with *R*^2^ = 0.68 and *R*^2^ = 0.62 (*p* < 0.0001). The results of the Reactome pathway analysis of the discriminative protein panel suggest that the molecular content of EVs might be indicative of tumor-specific biological processes.

**Conclusion:**

Integrating in vitro EV proteomic data, cell physiological characteristics, and clinical data of various tumor types illuminates the diagnostic, prognostic, and therapeutic potential of EVs.

Video Abstract

**Supplementary Information:**

The online version contains supplementary material available at 10.1186/s12964-023-01344-5.

## Background

Cancer growth, progression and metastasis are associated with genomic, proteomic, transcriptomic and metabolomic changes [1]. Omics sciences such as genomics, proteomics, transcriptomics and metabolomics are revolutionizing the understanding of cancer by comparing vast amounts of data with clinical features [2, 3]. Sources of data include in vitro experiments [4], clinical samples [5] and liquid biopsies [6], but nowadays extracellular vesicles (EVs) are of increasing interest due to their role in cell-to-cell communication, as they influence various physiological processes, including tumor-related functions such as immune regulation, cancer cell support, angiogenesis and metastasis [7–9].

Our research, along with others, suggests that EVs have great potential as a source of biomarkers that could advance the current state of cancer diagnosis because they provide a membrane-protected cargo that could reflect cell-specific pathological processes [10–14].

Numerous studies have highlighted the role of EVs in tumorous processes, leading to efforts to include them in liquid biopsy based diagnostic methods [15].

The majority of these studies have demonstrated that the analysis of EVs – due to the tumor-associated molecular pattern carried – can be used to differentiate between tumorous and control samples or to subcategorize tumor types based on their properties (e.g. chemosensitivity) [16–25].

However, there are still a number of unexplored areas regarding the potential utility of EVs. For instance, it is still under exploration whether the molecular composition of EVs can predict the invasion capacity or proliferation rate of the donor cells, or whether they could provide information on tumor-specific signaling pathways or strategies. Furthermore, as most of the studies investigate a limited number of groups, the degree of specificity of the molecular pattern carried by EVs of different tumor types is not fully elucidated.

Comprehensive studies of EVs derived from different tumor types are needed to fully explore their potential use in clinical practice. As a result, in recent years, there has been a rise in research into the proteome of EVs derived from the highly diverse NCI-60 cell line panel compiled by the National Cancer Institute. Using omics approaches to investigate the NCI-60 cell line panel, which contains 60 cell lines from nine tumor types, has significantly contributed to the discovery of potential biomarkers and drug targets, as well as understanding the molecular basis of chemotherapy resistance [26–40].

Beyond the research on the cell lysates, proteomic analysis of EVs of the NCI-60 cell lines revealed that their protein content reflects the molecular composition of the progenitor cell at both the proteomic and transcriptomic levels [41]. EVs were discovered to contain components of the core vesicle machinery, biomarkers already known from tissue, and integrin content that may be tumor stage-specific [41, 42].

Yet as omics and clinical data volumes rise, so do advances in information-processing tools, such as novel machine learning methods and advances in bioinformatics [43].

With this in mind, we hypothesized that we could mine valuable information on the role of EVs in tumor processes by comparing publicly available NCI-60 EV proteomics, cell physiology and clinical data using machine learning and the latest bioinformatics methods.

In our meta-analysis, we created classification models based on the entire proteome identified in the NCI-60 EVs as well as the proteins commonly identified in all samples. Using a selection algorithm, we compiled a panel of the most discriminative proteins from the entire proteome. Thereafter, we conducted enrichment analyses to determine which signal pathways our discriminative proteins are associated with these discriminative proteins. Furthermore, we assembled protein panels capable of estimating the invasion capacity and proliferation rate of donor cells, and validated them with in vivo clinical data.

## Materials and methods

### Data set used

#### Proteomic data

We obtained the proteomic data of EVs from the publication of Hurwitz et al. as freely downloadable supplementary material [41]. This data set contains the spectral count and intensity of 6,701 proteins for 60 EV isolates harvested from 60 cell lines (NCI-60) of nine different tumor types. In our study, we used the intensity values for the analyses. Before the analyses, the intensities were logarithmized in order to increase the linearity and reduce the variance. Imputation of missing values was not performed, as the 0 values in the data matrix used do not represent missing values, but the absence of proteins in the EV isolate.

#### Data on the invasion capacity of NCI-60 cell lines

The invasion phenotype of the 60 cell lines were obtained from the publication of DeLosh et al. as freely downloadable supplementary material [44].

Briefly, DeLosh et al. utilized CIM (cellular invasion/migration)-Plate 16 to determine the invasion capacity of the NCI-60 panel.

The CIM Plate-16 consists of two chambers, one below the other. The chambers are separated by a microporous membrane. Microelectronic sensors are integrated at the bottom of the pores in the lower chamber on the other side of the membrane. The migration of cells from the upper chamber to the lower chamber in response to a chemoattractant leads to their interaction and attachment to the electrical sensors, hence causing an elevation in impedance. The impedance correlates to increasing numbers of migrated cells on the underside of the membrane, and cell index values reflecting impedance changes are automatically and continuously recorded by the Roche xCELLigence Real-Time Cell Analyzer DP instrument. Therefore, cell migration activity can be monitored via the cell index profile.

The invasion phenotype of 60 cell lines was determined by plotting the cell index (reflecting the mass of the cell detected) as a function of analysis time and then calculating the area under the curve (AUC). We used the average AUC for each cell line as published in the original article, but refer to it as invasion capacity for ease of interpretation.

#### Data on the proliferation of NCI-60 cell lines

Doubling time of NCI-60 cell lines data were obtained from the National Cancer Institute website although [45], to facilitate interpretation, we refer to it as proliferation capacity for ease of interpretation.

#### Data on RNA expression of the NCI-60 cell lines

Microarray gene expression data was downloaded from the NCBI Gene Expression Omnibus (accession number: GSE32474) [46].

#### Data on the in situ tissue expression and survival data

In our study, we acquired information from the Human Protein Atlas database regarding the ex vivo tissue expression of specific proteins and the overall survival time (in years) of patients corresponding to the tissue samples [47].

### Classification of EV samples

During the classification, we attempted to classify the 60 EV samples into their respective nine tumor types (breast, central nervous system—CNS, colon, kidney, leukemia, lung, melanoma, ovary, prostate).

We applied multiple logistic regression on the proteomic data set for classification purposes.

First, the 60 EV sample was classified based on shared proteins and then on the entire proteome.

After classifying based on the entire proteome, we aimed to identify a discriminant protein panel for the nine tumor types.

The data set was split 50–50%, creating a Train and a Test set. We utilized the Least Absolute Shrinkage and Selection Operator (LASSO) method to score the proteins on the Train set according to their importance in distinguishing the tumor types (this score is the regression coefficients). This value can be negative, positive, or zero, suggesting a negative or positive effect on the probability of classifying into a certain tumor type, or an irrelevant protein.

In LASSO, the so-called cost strength parameter (C), which can vary from 0.001 to 1000, indicates how strict the scoring is (affecting the number of proteins scored as irrelevant/meaningless). In this study, this value was set to 1, which resulted in neither too strong nor too weak scoring, and allowed us to select characteristic proteins for each of the nine tumor types. The optimal value of the parameter C was determined by fivefold cross-validation of the train set and fixed at the point where the highest classification efficiency was measured.

The list of characteristic proteins for the nine tumor types included only proteins with a positive score obtained by LASSO. Classification was again performed on the Test data set based on the proteins selected.

The efficiency of the classification was given by the classification accuracy (number of correctly classified samples divided by the total number of samples). The success of the classification was visualized using confusion matrices.

Orange 3.27.0 [48] software was used to conduct the classification and create figures.

### Regression for invasion and proliferation capacity

To predict invasion and proliferation capacity, multiple linear regression with LASSO (with parameter C = 1) was performed. For regression, LASSO played the same role as in classification.

It should be noted that the approach (CIM Plate-16) used to determine invasiveness of the cell lines has been shown to be applicable only to solid tumors [44], therefore leukemia was not included in the determination of proteins predictive of invasion capacity.

During the procedure, the data was split 50–50%, creating a Train and Test set. On the Train set, LASSO was used to identify proteins that could potentially predict invasion and proliferation capacity. Then, using the Test set, the relationship between the selected proteins and invasion/proliferation capacity was investigated by multiple linear regression.

Value of *p* < 0.05 was considered significant.

The efficiency of the regression was given by the coefficient of determination (*R*^2^).

Orange 3.27.0, GraphPad Prism 8.4.3 (San Diego, CA, USA) were used for multiple linear regression and visualization.

### Pathway enrichment analysis

We utilized ShinyGO 0.76.3 for Gene Ontology Enrichment Analysis to determine the biological processes, molecular functions, and cellular components whose proteins are overrepresented in our data set [49]. The ShinyGO parameters were set to default.

Reactome (v82) was employed for simultaneous enrichment analysis of each sample in order to compare the 60 EV samples in terms of their associated signal pathways [50]. The Reactome parameters were set to default.

Value of *p* < 0.05 corrected with the false discovery rate (FDR) method was considered significant.

### Hierarchical clustering

Hierarchical clustering based on proteins was performed after row centering and unit variance scaling. Both rows (proteins) and columns (EV samples) were clustered using correlation distance and complete linkage.

Hierarchical clustering based on the Reactome results was performed on raw data, without any adjustment. The rows (pathways) were clustered using correlation distance and complete linkage.

Hierarchical clustering was performed using Morpheus software [51].

### T-distributed stochastic neighbor embedding

In order to visualize the proteomic data in a 2-dimensional space, we utilized the t-distributed stochastic neighbor embedding (t-SNE) method.

For t-SNE visualization, we used Orange 3.27.0.

### Examining the similarity between the EV proteome and the cellular RNA profile

The similarity of protein and RNA profiles of EV samples and cells for each variable was tested by Spearman’s correlation analysis, the results of which were plotted on heatmaps. In addition, the concordance of the two matrices (RNA profile of cells and protein content of EVs) was characterized overall with *R*_*V*_ coefficients introduced by Escoufier [52].

In data analysis, the *R*_*V*_ coefficient is a multivariate generalization of the squared correlation coefficient, depicting the similarity between two matrices of quantitative variables. The *R*_*V*_ coefficient takes values between 0 and 1.

The analysis was performed using the *omicade4* package in the R statistical framework [53].

### Survival analysis

The association between tissue expression of certain proteins and survival was determined by Kaplan–Meier analysis with logrank test, using GraphPad Prism 8.4.3. Value of *p* < 0.05 was considered significant.

## Results

### Machine learning methods revealed tumor-specific protein patterns of EV proteome

#### Shared proteins of EVs are related to EV biogenesis processes

The proteomic data set of the 60 EV samples contained 6,071 proteins. Intensity was measured for 5,908 proteins, referred to as the entire proteome in this study.

According to Gene Ontology Enrichment Analysis, the entire proteome is significantly associated with biological processes, molecular functions and cellular compartments such as neutrophil-mediated immunity, cell adhesion to the extracellular matrix, secretory vesicles and granules (Additional file 1). The fold enrichment values—which indicates how drastically genes of a certain pathway are overrepresented—ranged between 1.68 and 3.01. This means that we identified at least 1.68 times more proteins from the listed signal pathways as it would have been expected by chance.

Of the 5,908 proteins, 213 were present in all EV samples, referred to as the core proteome. The enrichment analysis of the core proteome showed that the shared proteins are involved in intracellular and EV biogenesis pathways, such as cotranslational protein targeting to membrane, RNA binding and cytosolic ribosomes (Additional file 2). Association of the core proteome with each biological pathway showed higher significance than the entire proteome, which was reflected in the fold enrichment values ranging from 3.78 to 33.12.

#### Entire proteome of EVs resulted higher classification accuracy of tumor cell lines than core proteome

We first inspected the core proteome for tumor-specific patterns using the logistic regression classification model.

**Fig. 1:**
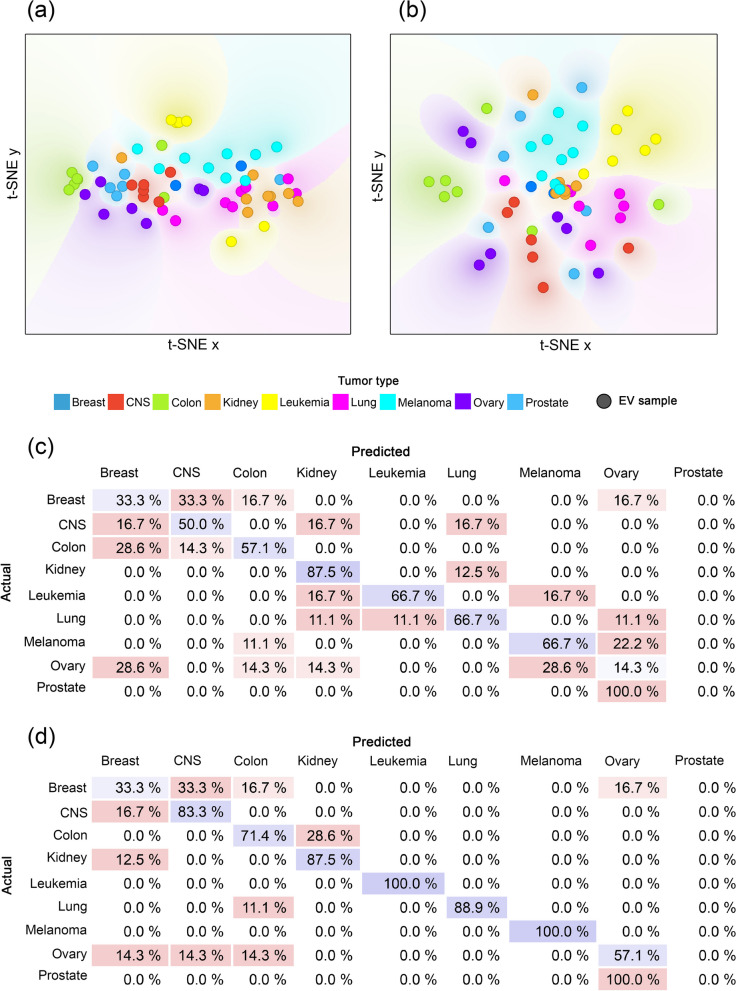
Classification efficiency based on the core and entire proteome. **a** t-SNE plot of the core proteome. **b** t-SNE plot of the entire proteome. The dots with different colors represent the 60 individual EV samples belonging to the nine tumor types. The color gradient in the plot indicates the dot density. **c** Confusion matrix of the classification results using the core proteome. **d** Confusion matrix of the classification results using the entire proteome. Each row of the matrices represents the instances in an actual class while each column represents the instances in a predicted class. Diagonally, the percentage of the correct classification is shown in blue. The percentage of errors is indicated in red

Remarkably, even this small subset of the entire proteome affecting a few biological processes carried enough specific information to distinguish certain tumor types from the others to some extent, such as kidney, lung, leukemia and melanoma (Fig. 1a, c). The classification accuracy of 49.14% significantly outperformed the 11.1% that would have been obtained with random classification.

As expected, a one-way ANOVA analysis revealed that the average intensity of the core proteome depends on tumor type (*p* < 0.0001). However, Pearson’s correlation analyses confirmed that this difference could not be caused by differences in EV secretion, EV mean and mode size, or cell size. No significant correlation was identified between any parameter and the average intensity of the core proteome. This suggests that the unique core proteome pattern is not caused by the difference in EV production rate and type of EVs between the nine tumor types, but the different tissue origin.

Using the entire proteome, the distinction between tumor types had become even more defined (Fig. 1b, d). Classification accuracy significantly increased for CNS, colon, leukemia, lung, melanoma, and ovary. The average classification accuracy increased to 69.10% which is 57.99% higher than chance.

#### The EV proteome could be used to form a discriminative protein panel

In exploring the discriminatory protein panel, we have taken care to ensure that the method does not become overestimated or overfitted. To achieve this, the 60 cell lines were split 50–50%. On one half of the cell lines, the Train set, we applied the LASSO algorithm.

Using the LASSO method, we were able to assign importance scores to each protein of the entire proteome based on their ability to differentiate the nine tumor types in the Train set. The selection algorithm (with parameter C = 1) resulted in 172 proteins, which were further investigated for hierarchical clustering, classification purposes and Reactome pathway analysis (Additional file 3).

In the hierarchical clustering, the Train and Test sets were analyzed together on the basis of 172 proteins.

**Fig. 2:**
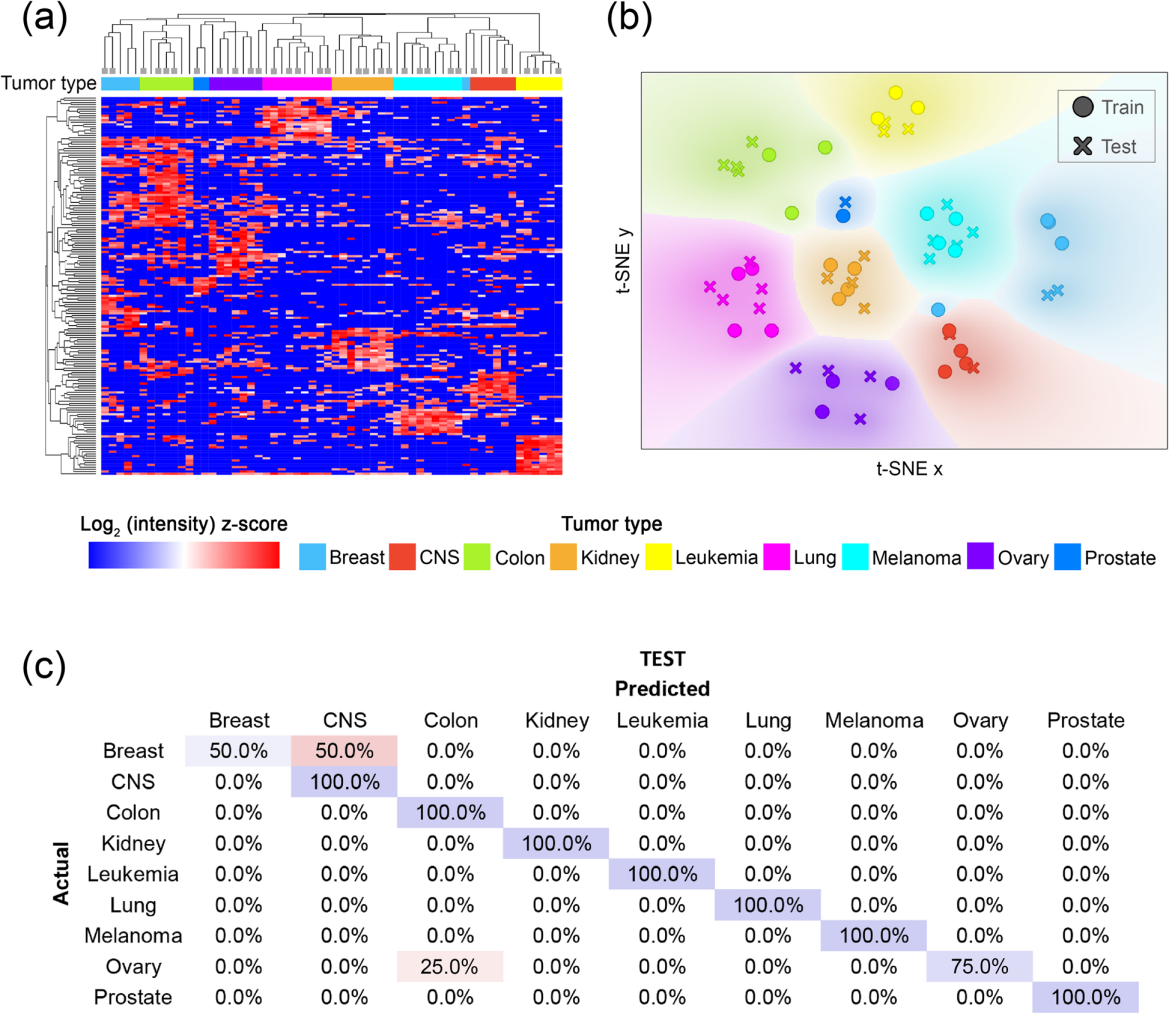
Classification efficiency for the selected proteins. **a** Heatmap with hierarchical clustering. In the heatmap, the columns and rows represent the 60 EV samples belonging to the nine tumor types marked with different colors and the 172 proteins, respectively. Both the columns and rows are clustered. Dendrogram branches ending in a square indicate the elements to be included in the Train set. **b** t-SNE plot of the selected 172 proteins. The dots with different colors represent the 60 individual EV samples belonging to the nine tumor types. In the plot, the color gradient indicates the dot density. **c** Confusion matrix of the classification results using the selected proteins on the Test set. Each row of the matrices represents the instances in an actual class while each column represents the instances in a predicted class. Diagonally, the percentage of the correct classification is shown in blue. The percentage of errors is indicated in red

Hierarchical clustering using a heatmap revealed that the 172 proteins form a well-defined pattern, enabling the 60 EV samples to form nearly perfectly homogenous clusters, while the Train and Test sets elements are clustered together (Fig. 2a).

This separation is also evident in the t-SNE plots, which depict the various tumor types as distinct groups (Fig. 2b). Again, the elements of the Train and Test sets populated the same areas.

When the samples of the Test set were classified based on the 172 proteins, an average classification efficiency of 91.67% was achieved (Fig. 2c).

For the whole data set (Train + Test), the average efficiency was 96.60%.

### Discriminative proteins might uncover tumor-specific pathways

**Fig. 3:**
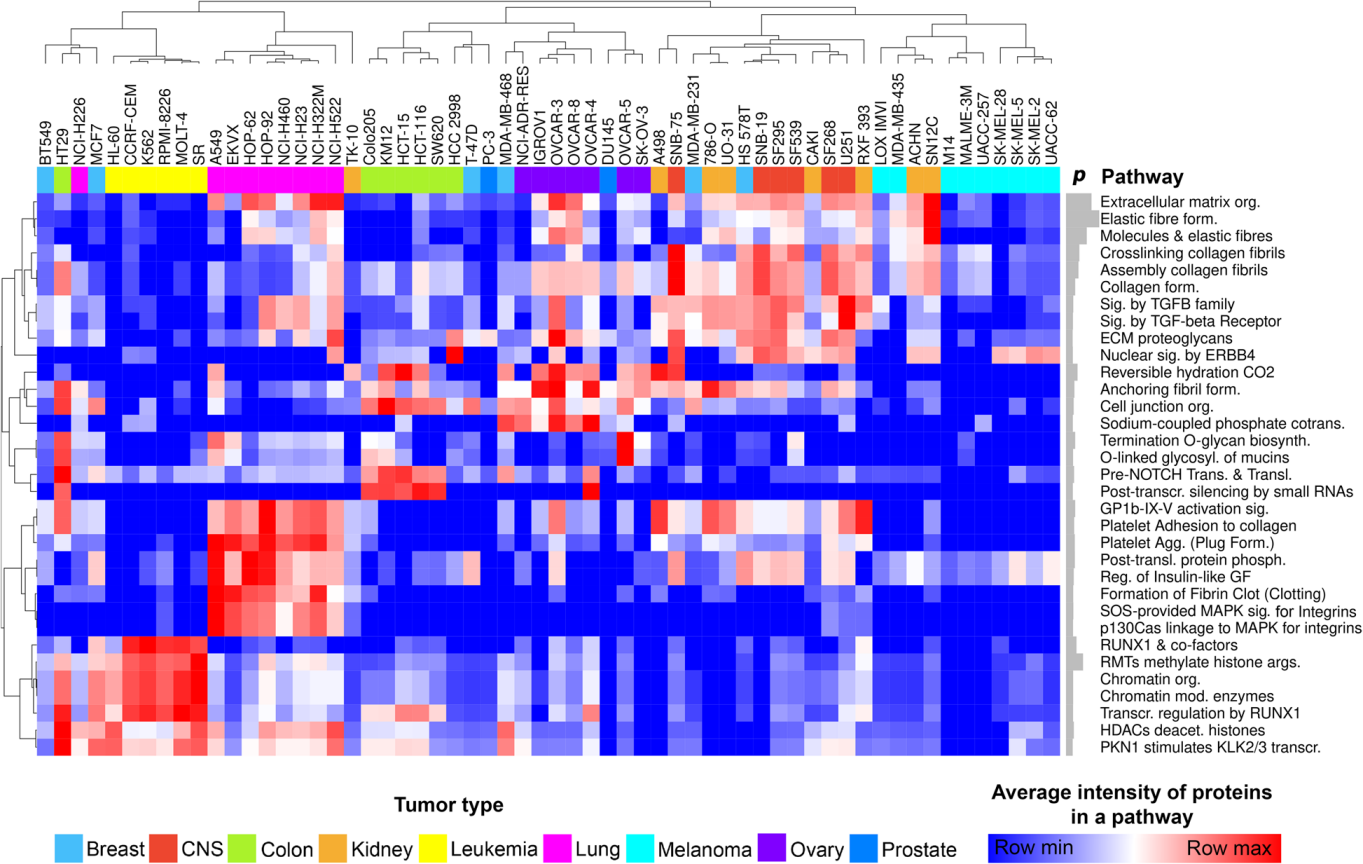
Biological signaling pathways affected by the 172 selected proteins of the discriminative protein panel. The columns marked with different colors represent the 60 EV samples, while the rows indicate the various signaling pathways. Both the 60 samples and pathways were clustered hierarchically. The heatmap values represent the average intensity of the proteins that are part of a given signal pathway. The gray barplots next to the names of the pathways indicate the -log_10_(*p* value). In all instances, *p* < 0.05. (agg.: aggregation; biosynth.: biosynthesis; cotrans.: cotransporters; deacet.: deacetylate; form.: formation; mod.: modifying; org.: organization; phosph.: phosphorylation; prots.: proteoglycans; sig.: signaling; trans.: transcription; transl.: translocation)

After selecting the proteins, we hypothesized that – given the proteins' large intergroup differences – the biological signaling pathways they affect would also exhibit distinctive patterns. In order to place the 172 selected proteins in a biological context Reactome enrichment analysis was utilized. Only those pathways with *p* < 0.05 were considered for hierarchical clustering and heatmap creation (Fig. 3).

The selected 172 proteins are associated with extracellular matrix, nuclear processes, and cell division-related signaling pathways.

Although cancers of the breast and prostate lacked characteristic signaling pathways, the majority of the EV samples clustered according to their tumor type revealing a distinctive signaling pathway pattern.

The collagen matrix, TGF-β receptor, and ERB4 enzyme signaling pathways were identified as common characteristics for both kidney and central nervous system tumors, which clustered together.

Compared to other tumors, leukemia samples exhibit a predominance of nuclear processes associated with histone and chromatin modification.

In general, lung tumors were distinguished by platelet-associated biological processes and integrin-signaling pathways.

### Extracellular vesicles carry information on invasion and proliferation capacity

The NCI-60 cell line panel contains not only tumors of different tissue origin, but also tumors with different invasion capacities and different division rates.

Noting that tumor cell lines with low invasion capacity such as BT549 and Hs 578 T (breast) were classified into tumors with high invasion capacity (e.g. CNS) during classification and hierarchical clustering the question arose whether further protein panels predicting invasion and proliferation capacity could be defined.

To construct a panel correlated with invasion and proliferation capacity, multiple linear regression with LASSO selection method was utilized.

As in the classification procedure, the data set was split 50–50%. On the Train set, we used LASSO to identify proteins that could be predictive for invasion capacity and proliferation, then validated the findings on the Test set.

The selection resulted in 20 and 15 proteins, which tended to have predictive potential for invasion and proliferation capacity in the Train set, respectively (invasion panel and proliferation panel).

The Test set was then used to validate the predictive value of the panels using multiple linear regression.

**Fig. 4:**
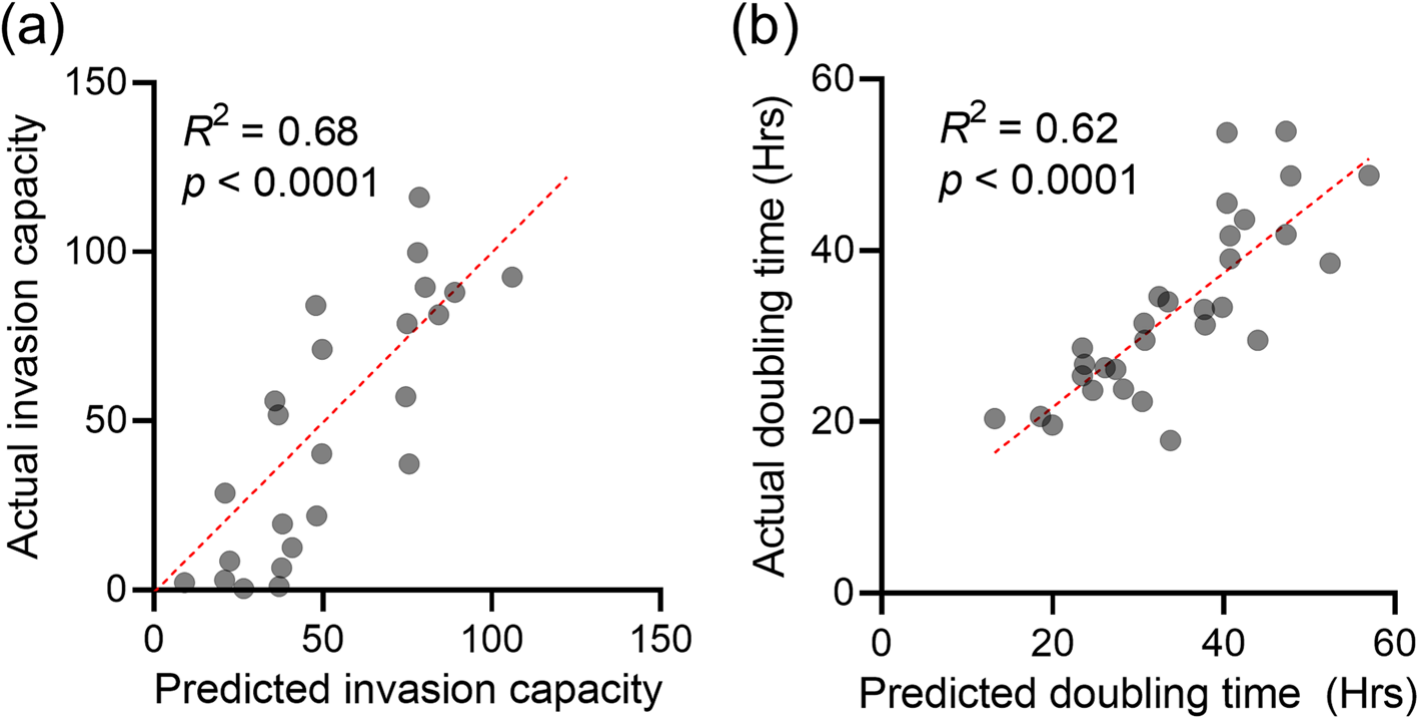
Results of the multiple linear regression. **a** Multiple linear regression of invasion capacity. The invasion capacity predicted by the invasion panel for each sample in the Test set is plotted on the x-axis, while the actual invasion capacity is plotted on the y-axis. **b** Multiple linear regression of proliferation capacity. The doubling time predicted by the invasion panel for each sample in the Test set is plotted on the x-axis, while the actual doubling time is plotted on the y-axis. (*R*^2^—coefficient of determination; *p*—*p* value.)

Multiple linear regression showing significant results for both the invasion panel and the proliferation panel (*p* < 0.0001), we also obtained remarkably high coefficients of determination: *R*^2^ = 0.68 for the invasion, *R*^2^ = 0.62 for the proliferation capacity (Fig. 4). Pooling the Test and Train sets, the *R*^2^ values were found to be 0.71 and 0.69, respectively.

**Fig. 5:**
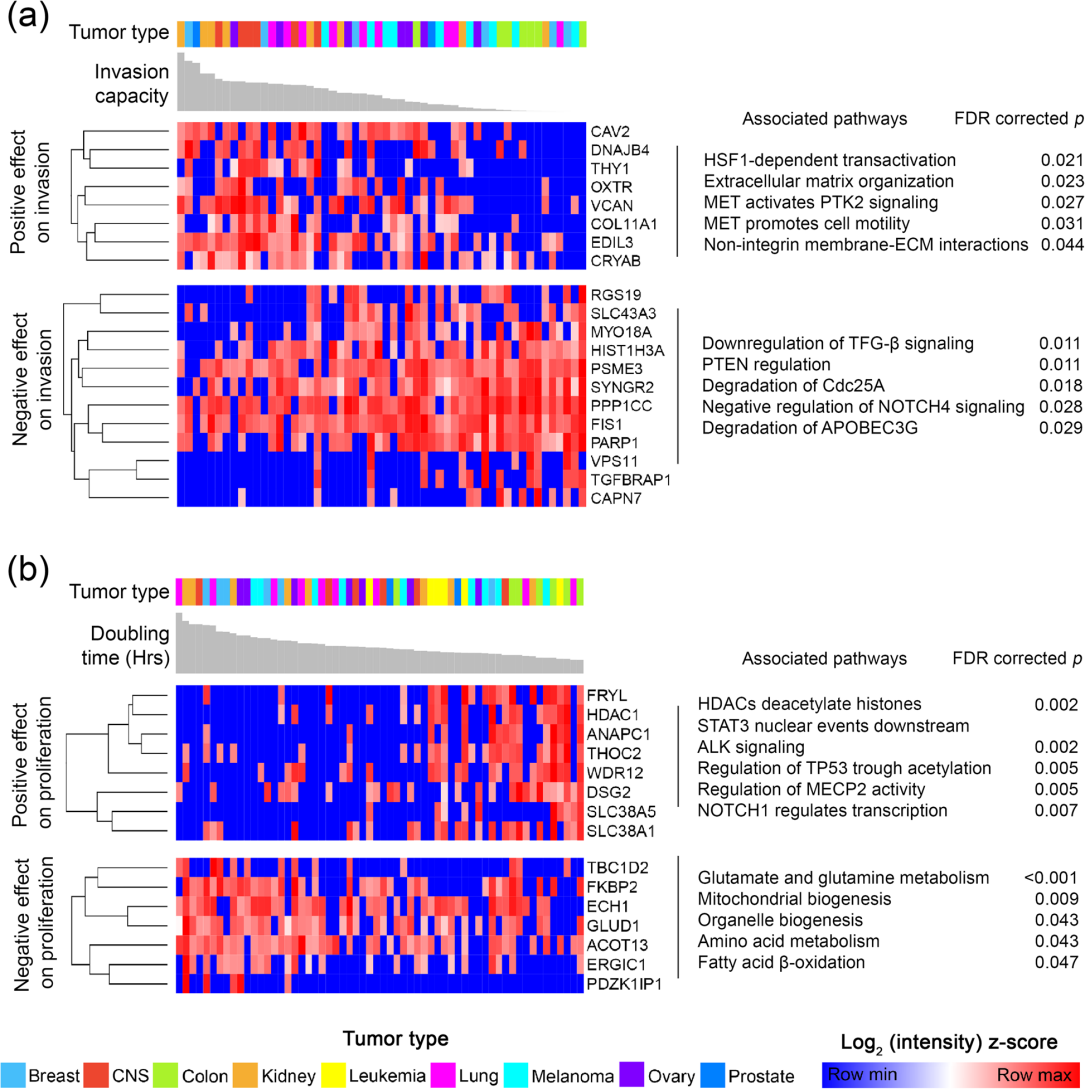
Predictive proteins for invasion and proliferation capacity. **a** Predictive protein panel for invasion capacity (invasion panel). The columns marked with different colors and the gray barplots indicate the 54 EV samples with the invasion capacity measured for the cell line of origin (leukemia not included). The rows indicate the proteins, which were clustered hierarchically. Two defined clusters were separated from each other. **b** Predictive protein panel for proliferation capacity (proliferation panel). The columns marked with different colors and the gray barplots indicate the 60 EV samples with the doubling time (in hours) measured for the cell line of origin. The rows indicate the proteins, which were clustered hierarchically. Two defined clusters were separated from each other. It should be noted that higher doubling time means lower proliferation capacity as it indicates more time for cell division

After validation on the Test set confirmed the predictive value of the proteins, both of the 20- and 15-member panels (Additional file 4) were then subjected to hierarchical clustering, which resulted in 2–2 clusters (Fig. 5): one cluster that appears to be negatively correlated and another that appears to be positively correlated with invasion or proliferation capacity.

Of the 20-member invasion panel, eight proteins (CAV2, DNAJB4, THY1, OXTR, VCAN, COL11A1, EDIL3, CRYAB) positively predicted the invasion capacity of the cell lines. Based on Reactome pathway analysis, these proteins were significantly associated with signaling pathways that upregulate tumor cell maintenance, invasion and binding to the extracellular matrix. Similarly, the enrichment analysis of the remaining twelve proteins that negatively predict invasion capacity was consistent with the regression results: these proteins play a role in pathways that negatively regulate the invasion (Fig. 5a).

The eight proteins that positively influence proliferation capacity were associated with processes linked to cell cycle. While seven proteins negatively associated with proliferation are linked to metabolic pathways (Fig. 5b).

We further attempted to gain more support for our invasion and proliferation capacity prediction panels by examining their impact on patients’ survival time.

The Human Protein Atlas (HPA) was considered an appropriate database for this purpose, as it contains survival times for a large number of cancer patients for all nine cancer types and is easily accessible. However, we had to take into account the limitation that HPA contains tissue RNA expression data and not EV proteomic data.

Accordingly, before utilizing the HPA database, we had to assess the similarity of EV protein and cellular RNA patterns to be permitted to investigate the effect of in vivo RNA tissue expression of panel members on survival time.

**Fig. 6:**
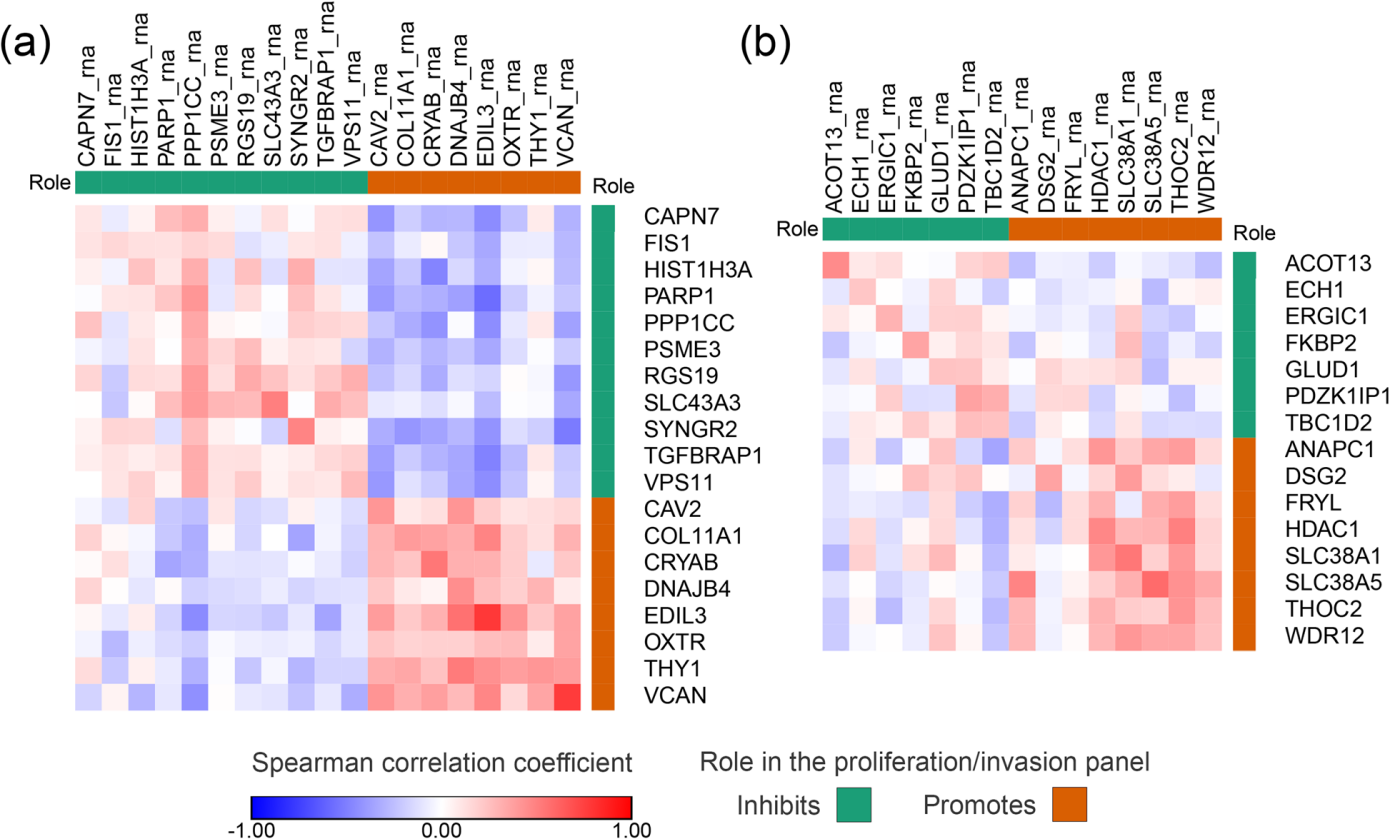
Correlation of EV protein and cellular RNA content. The heatmaps show the correlation between cellular RNAs and EV proteins of invasion (**a**) and proliferation (**b**) panel members. Columns represent the cellular RNA, rows represent the EV proteins

First, we examined how the EV protein panels (invasion and proliferation) and the cellular RNAs correlate with each other (Fig. 6). Based on the results, the RNA and protein patterns of the invasion panel showed a moderately strong concordance (*R*_*V*_ = 0.51, *p* = 0.020). While a weaker but still significant relationship was observed when comparing the RNA and protein matrices of the proliferation panel (*R*_*V*_ = 0.39, *p* = 0.048). Notably, we observed stronger pairwise correlations between protein and RNA content for the promoting members of both panels.

After assessing the relationship between EV protein and cellular RNA pattern, we attempted to use the cellular RNA to estimate the invasion and proliferation capacity of cells using the panel members.

Based on the cellular RNA, invasion capacity could be estimated at *R*^*2*^ = 0.77 (p < 0.0001) and proliferation capacity at *R*^*2*^ = 0.32 (*p* = 0.037).

The in vitro data suggested that the EV proteomic and cellular RNA patterns are in concordance and that the cellular RNA content is also related to invasion and proliferation capacity in a similar way as the EV proteome. This prompted us to investigate the impact of in vivo RNA tissue expression of panel members on patient survival.

Using the HPA database, we collected clinical data on the tissue expression of our panel members in the nine tumor types from 4,665 patients, then examined the relationship between tissue expression and 5-year survival rate.

In the HPA database, tissue expression was found for 19 of the 20 proteins of the invasion panel (Additional file 5).

**Fig. 7:**
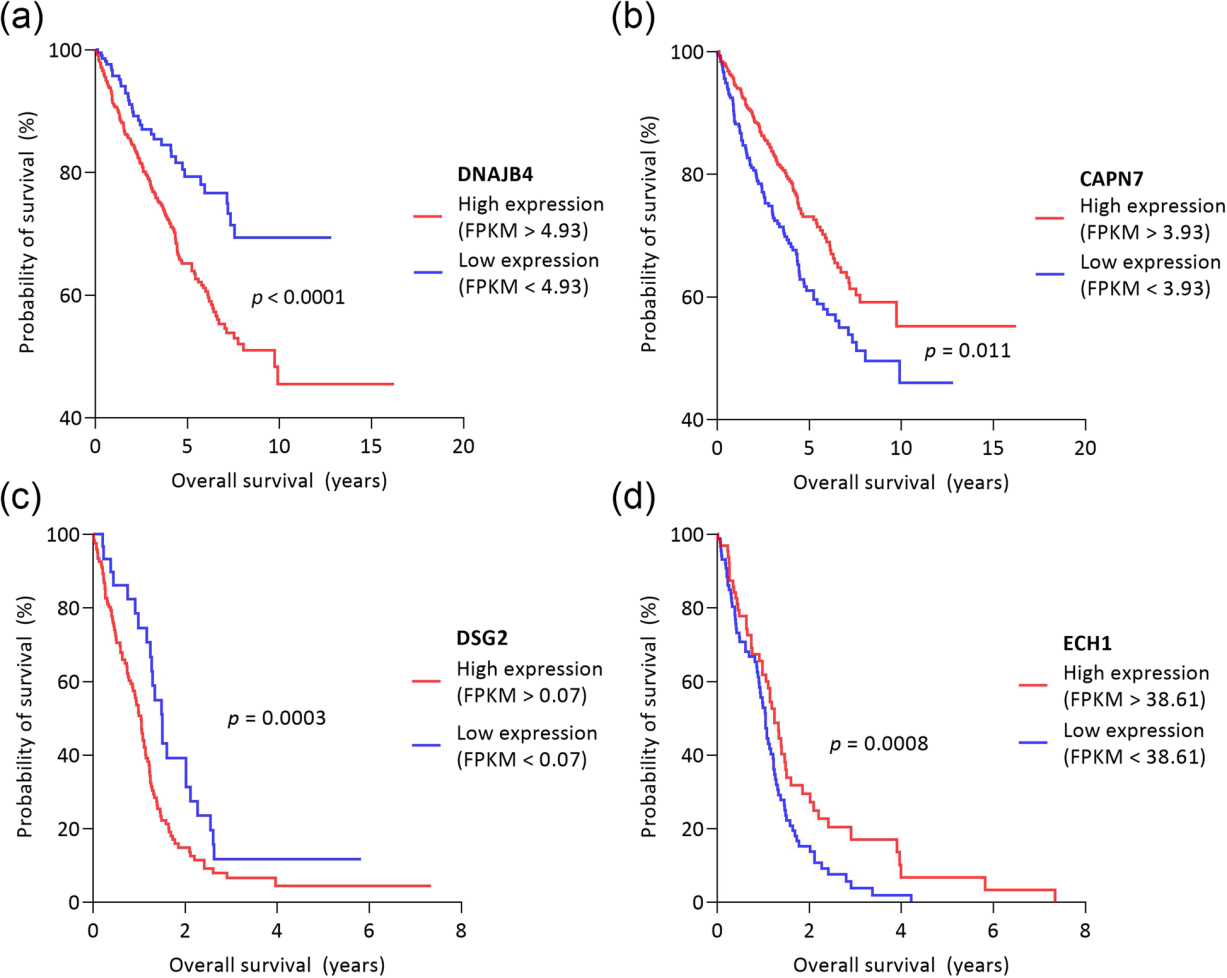
Survival functions for different expression levels of DNAJB4, CAPN7, DSG2, ECH1. The figure shows 4 exemplary proteins selected from the members of the invasion and proliferation panel and their impact on patients’ survival. **a** DNAJB4, which we found to be positively associated with invasion and which the Human Protein Atlas (HPA) suggests that its high expression is associated with a worse prognosis in kidney tumors (*n* = 877). **b** CAPN7 protein, which in our study is negatively associated with invasion and which the HPA suggests may be associated with a favorable prognosis in kidney tumors. **c** DSG2 protein which in our study positively predicted the proliferation capacity is a negative prognostic factor in CNS tumors, based on HPA. **d** Based on our results, ECH1 protein negatively predicted the proliferation capacity, and it is a favorable prognostic marker for CNS tumors

According to the HPA, high expression of CAV2, COL11A1, DNAJB4, THY1 and VCAN decreased the 5-year survival for breast, CNS, colon, kidney, lung and ovarian tumors (Fig. 7a). These findings are in line with our results, as these proteins were found to be positively associated with invasion capacity according to multiple linear regression analysis.

The CRYAB protein was found to be controversial, as our results showed a positive association with invasion, but in HPA, high tissue expression was associated with a better prognosis in CNS tumors. Nevertheless, in colon tumors, high expression was a negative prognostic marker.

The case is similar for EDIL3, which is positively associated with invasion capacity according to multiple linear regression analysis, but based on the HPA, higher tissue expression is associated with better 5-year survival in colon tumors. However, it still was a significantly worse prognostic marker in breast, kidney and melanoma patients.

Overall, the effects on survival found in the HPA database and the effect of the proteins on invasion capacity as determined in our study were consistent in 90% of the cases.

Based on multiple linear regression, twelve proteins in our study were found to be negatively correlated with invasion capacity. Comparing this finding to the HPA database, we found more inconsistencies: according to the HPA, the twelve proteins are favored prognostic markers for 5-year survival in most cases (73.18%) (Fig. 7b), but in 26.82%, the proteins have an adverse effect on survival than the expected. For example, HIST1H3A showed a negative association with invasiveness in our study, but its high expression negatively affected the survival rate of CNS tumor patients according to the HPA database (Additional file 5).

Tissue expression was found for all the 15 proteins of the proliferation panel (Additional file 6). The proliferation panel contains seven proteins which were found to negatively predict the proliferation capacity. According to HPA, high tissue expression of these seven proteins significantly increased the 5-year survival in 64.71% of cases (Fig. 7c). Vice versa, the high expression of the eight proteins which positively predict the proliferation capacity significantly reduces the 5-year survival in 72.41% of cases (Fig. 7d).

Taken as a whole, the EV proteome and in vitro cellular RNA pattern of the panel members showed concordance, and the effect of in vivo tissue RNA expression of the panel members on patient survival is consistent with the results of our linear regression model. The finding potentially suggests the involvement of invasion and proliferation panels in the tumorous processes.

It is noteworthy that the inconsistency with HPA appears for those variables where the in vitro EV proteome and cellular RNA pattern did not show a strong correlation (invasion capacity inhibitory members) (Fig. 4), or cellular RNA did not prove to be a sufficient predictor (overall the proliferation panel).

## Discussion

Nowadays, EVs are considered as a novel and promising tool for liquid biopsy-based cancer diagnosis, prognosis and therapeutic decisions. However, there are barely explored segments of their potential clinical applicability.

In the present study, we aimed to determine the degree of specificity of the proteome carried by EVs from various tumor types, as well as whether the EVs’ molecular pattern can be used to predict the invasion capacity and proliferation rate of the donor cells.

In our meta-analyses, we investigated the proteome of EVs isolated from the supernatant of NCI-60 cell lines. Of the total proteome, 213 proteins were present in all EV samples (core proteome). Although these proteins were observed in all tumors, they showed some degree of specificity.

Based on Gene Ontology Enrichment Analysis, these protein sets are associated with biological pathways, molecular functions, and cellular components including protein targeting, cotranslational modifications, RNA binding and processing, ribosomal subunit, and exocytotic pathways. These findings are consistent with those previously described by Hurwitz et al. [41, 54]. As it has been pointed out before, this enrichment may indicate that the core proteome facilitates cell-to-cell communication by directly translating the mRNA content of EVs following fusion with the target cell.

Even though the core proteome showed differences between the nine tumor types, the reason for these differences could not be determined from the available data. Our correlation analyses suggested that the distinct core proteome pattern was not caused by the difference of EV production rates or EV type between the nine tumor types. Therefore, we assumed that the source of the observed variance in the core proteome is the different origin of the nine tumor types.

Extending the analysis to the entire proteome, then to the selected protein set significantly improved classification accuracy, indicating that the molecular signature carried by EVs is remarkably characteristic of certain tumor types, and this specificity could be further increased by using the appropriate selection methods.

This finding is in accordance with previous literature data. However, most studies have attempted to distinguish between cancerous samples and matched controls, or to subcategorize different tumor types in both in vivo and in vitro experiments [16].

For example, by selecting the proteins detected in EVs, Vinik et al. showed that the control and breast cancer patient groups were significantly distinguishable from each other [17].

The diagnostic efficacy of vesicles has also been demonstrated for brain tumors. In an in vivo experiment with mice, Anastasi et al. used principal component analysis to show that the proteome of control and mice with glioblastoma multiforme differed significantly [18].

Moreover, diagnostic importance has also been reported for ovarian, colon cancer and leukemia [19–21].

In addition to distinguishing a tumor cohort from a matched control sample, studies can be found about stratifying a cancerous disease according to different characteristics. For example, Li et al. investigated plasma EVs to highlight leukemia patient groups with different imatinib resistance [22]. Choi et al. distinguished between primary and metastatic colon tumors [23]. Mallawaaratchy et al. identified glioblastoma subtypes of aggressiveness [24], and Rontogianni et al. pointed out that proteomic analysis of EVs allows the differentiation of breast cancer subtypes [25].

Our study differs from these in that our aim was not to investigate the differences from control samples or to subcategorize a certain tumor, but to distinguish a wide range of tumors with different tissue origin. In a well-written article, which was the source of the NCI-60 proteomic data set Hurwitz et al. have already demonstrated that some tumor types are distinguishable from the others [41].

Approaching this valuable dataset with the evolving machine learning based classifier algorithms suggests that the proteomic content carried by cancer EVs is more specific than expected and previously reported.

Uncovering tumor-specific signaling pathways is a key element in identifying drug targets [55]. Most research focuses on the analysis of tissue, however, obtaining tissue biopsy from certain tumors, particularly brain tumors, carries high risks for the patient, has limited reproducibility, and does not provide reliable information due to intratumoral heterogeneity [56]. However, these challenges can be overcome by using EVs isolated from the circulation, as their molecular content provides information about the entire tumorous condition [57].

Although there is a growing body of research on the use of EVs as drug carriers, no studies have investigated the molecular content of EVs in an attempt to identify drug targets [58].

Our results suggest that the proteins showing the largest group differences between the nine tumor types may indicate tumor type-specific signaling pathways and specific strategies.

For example, matrix-related processes were proven to be specifically involved in CNS and kidney tumors. Pointer et al. have shown that collagen matrix structure plays a significant role in the survival of patients with glioblastoma: the presence of disorganized fibers is associated with a significantly worse prognosis [59]. Similar results have been described in kidney cancer, where collagen matrix structure predicted the tumor grade [60].

NOTCH signaling was found to be specifically characteristic for colon cancers based on the EV proteome. Consistent with our findings, several studies have highlighted that NOTCH signaling is essential for the initiation of colon cancer cell development [61].

We also found a strong association between the leukemia EV proteome and processes associated with the transcription factor RUNX1, whose mutation has been shown to play an important role in the development of hematological malignancies [62].

In addition to the above examples, the results of our enrichment study are supported by further literature on leukemia [63], melanoma [64], lung [65, 66] and ovarian cancer [67, 68].

Extending and applying our knowledge on the invasiveness and proliferation rate of cancer cells is vital for the proper treatment and prognosis of patients. In estimating patient survival, the number of metastatic nodules and the size of the tumor mass are particularly crucial variables [69–72].

Our findings suggest that the EV proteome can provide information about the donor cells’ proliferation rate, and invasion capacity, which are crucial steps in tumor progression and metastasis formation [73].

The predictive invasion and proliferation panel were subjected to Reactome pathway analysis to reveal the physiological mechanisms of the predicted effects. For instance, we found that EV proteins detected in high invasion capacity tumor cell lines may induce HSF1-dependent transactivation. This finding is supported by literature data; amplification of HSF1 was shown in a wide variety of tumors with a 10.33–26.54% alteration frequency in the most aggressive tumors, i.e. ovarian epithelial tumors, breast cancer, pancreatic cancer [74, 75].

As HSF-1 is a main transactivator of HSPs expression, including HSP60, HSP70, and HSP90, it has multiple effects on cancer progression, such as promoting invasion and metastasis [76].

Our data show that proteins predicting low invasion may cause downregulation of TGF-β signaling. Indeed, TGF-β may function as a tumor promoter by stimulating epithelial-mesenchymal transition (EMT) of tumor cells leading to metastasis [77]. Also, inactivation of TGF-β signaling suppress prostate cancer bone metastasis [78].

Panel members, which positively predict proliferation capacity are significantly associated with reversible histone acetylation by HDAC enzymes. Several studies have investigated HDAC and proliferation; for example, HDAC enzymes are important in melanoma tumor cell proliferation [79]. And again, inhibition of HDACs represses proliferation of head and neck squamous cell carcinoma cells [80]. In addition, various phases of preclinical trials are addressing the inhibition of HDAC in subjects with mutated advanced and unrespectable melanoma (ClinicalTrials.gov ID: NCT02836548, NCT02032810).

From the list of proteins which are associated with lower proliferation, the GLUD1 (glutamate dehydrogenase 1) were shown to influence glutamate and glutamine metabolism. It is evidenced so far that glutamine metabolism enhances the proliferation and tumor growth [76]. However, high expression of GLUD1 may predict good overall patient outcome [81]. Coloff et al. showed negative correlation between GLUD1 and proliferation, concluding that highly proliferative tumors couple glutamine anaplerosis to non-essential amino acid synthesis [82].

Despite the fact that the results of the meta-analysis appear to be supported by other findings, it is important to draw attention to the limitations of our work.

The data set is relatively small compared to the number of elements required for machine learning: it contains proteomic data from EV samples of 60 cell lines, and the nine tumor types have different sample numbers.

However, we found that even 50% of the data was enough for the Train set to learn important patterns from the data that could be applied to the Test set. We believe that despite the small number of elements, we could find generalizable differences. Nevertheless, we acknowledge the importance of validating the findings on a larger dataset to ensure the robustness of the results.

Hurwitz et al. described a strong correlation between the proteomic pattern of EVs and the cellular RNA content [41]. Our study has highlighted that within the entire proteome, our invasion and proliferation panels are also in concordance with the cellular RNA pattern. This finding prompted us to investigate the impact of in vivo RNA expression of panel members on tumor patient survival.

The predictive value of the invasion and proliferation panel established in this study was supported by the literature and the Human Proteome Atlas (HPA) database. Nevertheless, the authors acknowledge and strongly emphasize that comparing the in vitro EV proteome and in vivo tissue RNA expression is an implicit approach even if the relationship between the EV proteome and the in vitro cellular RNA pattern has been successfully assessed. The comparison is not intended to validate the panel members, but rather to suggest potential biomarker targets that may be worthy of further research.

The main limitation of the study is that its results are based on 2D in vitro data. 2D cultures have several limitations, such as perturbation of interactions between the cellular and extracellular environment, changes in cell morphology, polarity and proliferation mode [83]. The authors certainly acknowledge the need for further validation, and consider the results presented here only as promising research candidates, not as an unimprovable approach to the in vivo phenomenon.

A previous meta-analysis has already analyzed the proteome of NCI-60 EVs, but with different assumptions [84]. In this research, the investigation aimed to determine the potential support of EV proteomes in facilitating the functional transfer of cancer hallmarks. The study conducted a meta-analysis, where a comparison was made between EVs and entire cell proteomes derived from the NCI-60 cell lines. A distinct subset of proteins within each cancer hallmark signature was identified, demonstrating both high abundance and consistent expression within EVs across all cell lines.

To our knowledge, ours is the first study to classify such a large number of tumor types based on proteomic data from EVs, looking for discriminative patterns, and to investigate the predictive value for donor cell invasion capacity and proliferation rate using machine learning techniques, which could greatly help in evaluating the potential clinical applications of EVs.

## Conclusions

Our results suggest that the extensive body of knowledge on EV omics research to date is worth re-exploring with the emerging and increasingly available state-of-the-art methods. Integrating proteomic data from EVs from different tumor types with cell physiological and clinical data can help to reveal the full potential of EVs in oncology. By studying their molecular content, it may be possible to obtain information on tumor properties that are crucial for patient treatment, such as invasion and proliferation capacity. In addition, they may also allow us to unravel the signaling pathways and biological processes underlying the specific characteristics of different tumor types, helping to identify potential drug targets.

### Supplementary Information

**Additional file 1.** Gene Ontology Enrichment for the entire proteome.

**Additional file 2.** Gene Ontology Enrichment for the core proteome.

**Additional file 3.** The selected 172 proteins.

**Additional file 4.** Members of the invasion and proliferation panels.

**Additional file 5.** Comparison of the invasion panel with the Human Protein Atlas database.

**Additional file 6.** Comparison of the proliferation capacity panel with the Human Protein Atlas database.

## Data Availability

All data generated or analyzed during this study are included in this published article and its supplementary information files.

## Abbreviations

ANOVA: Analysis of variance
AUC: Area Under the Curve
CAPN7: Calpain 7
CAV2: Caveolin 2
CNS: Central nervous system
COL11A1: Collagen type XI alpha 1 chain
CRYAB: Alpha-crystallin B
DNAJB4: DnaJ heat shock protein family (Hsp40) member B4
DSG2: Desmoglein 2
ECH1: Delta(3,5)-Delta(2,4)-dienoyl-CoA isomerase
EDIL3: EGF like repeats and discoidin domains 3
ERB4: Erb-b2 receptor tyrosine kinase 4
EV: Extracellular vesicle
FDR: False discovery rate
GLUD1: Glutamate dehydrogenase 1,
HDAC: Histone deacetylases
HIST1H3A: Histone H3.1
HPA: Human Proteome Atlas
HSF1: Heat shock transcription factor 1
HSP: Heat shock protein
LASSO: Least Absolute Shrinkage and Selection Operator
mRNA: Messenger ribonucleic acid
NCI-60: National Cancer Institute 60
NOTCH: Neurogenic locus notch homolog
OXTR: Oxytocin receptor
*p*: *p* value
PCA: Principal component analysis
*R*^2^: Coefficient of determination
RNA: Ribonucleic acid
RUNX1: RUNX family transcription factor 1
TGF-β: Transforming growth factor beta
THY1: Thy-1 cell surface antigen
t-SNE: T-distributed stochastic neighbor embedding
VCAN: Versican
